# Food allergy and anaphylaxis – 2047. Anaphylaxis to topically applied sodium fusidate on abrasions

**DOI:** 10.1186/1939-4551-6-S1-P132

**Published:** 2013-04-23

**Authors:** Kangmo Ahn

**Affiliations:** 1 Pediatrics, Samsung Medical Center, Sungkyunkwan University, Seoul, South Korea

## Background

Fusidic acid is a bacteriostatic antibiotic that is effective primarily on gram-positive bacteria such as Staphylococcus species and Corynebacterium species. It is often used topically on skin, but also is given systemically as tablets or injections. Allergic contact dermatitis or urticaria has been reported as side effect of fusidic acid, but anaphylaxis to topically administered fusidic acid have not been reported yet.

## Methods

Clinical history was throughly assessed, and oral challenge test was done to confirm the drug-induced anaphylaxis.

## Results

A 16-year-old boy visited outpatient clinic due to further evaluation of anaphylaxis. He suffered abrasions on his arms during exercise and was treated with topical ointment containing sodium fusidate on the abrasions. Within 30 minutes, he developed urticaria and eyelid swelling, followed by cough and respiratory difficulty. His symptoms were relieved by emergency treatment in nearby hospital. In order to investigate the etiology, oral provocation with fusidate was performed. After 125 mg (1/2 tablet) of sodium fusidate was administered, he developed cough and itching of the throat in 30 min, followed by chest discomfort and urticaria. Forced expiratory volume in 1 second (FEV_1_) dropped from 4.09 L at baseline to 3.50 L after challenge, although wheezing was not heard over his chest. After management with inhaled bronchodilator via nebulizer, chest discomfort was relieved and FEV_1_ rose to 3.86 L.He was recommended not to use fusidate especially on the abrasions.

## Conclusions

Herein we report the first case of anaphylaxis by topical fusidic acid on the abrasions.

